# Diagnostic methods and protocols for rapid determination of methicillin resistance in *Staphylococcus aureus* bloodstream infections: a comparative analysis

**DOI:** 10.1007/s10096-025-05039-2

**Published:** 2025-01-22

**Authors:** Matteo Boattini, Luisa Guarrasi, Sara Comini, Guido Ricciardelli, Roberto Casale, Rossana Cavallo, Cristina Costa, Gabriele Bianco

**Affiliations:** 1 Department of Public Health and Paediatrics, University of Torino, Turin, Italy; 2 Microbiology and Virology Unit, University Hospital Città Della Salute E Della Scienza Di Torino, Turin, Italy; 3 Lisbon Academic Medical Centre, Lisbon, Portugal; 4 Operative Unit of Clinical Pathology, Carlo Urbani Hospital, Ancona, Italy; 5 Department of Experimental Medicine, University of Salento, Lecce, Italy

**Keywords:** MRSA, *S. aureus*, Eazyplex® MRSA plus, MALDI-TOF MS subtyping, Clearview™ PBP2a SA Culture Colony test, Immunochromatography, EUCAST RAST

## Abstract

**Purpose:**

To evaluate diagnostic performance of four diagnostic methods for rapid determination of methicillin resistance in *S. aureus* positive blood cultures (BCs).

**Methods:**

Clinical and spiked BCs were subjected to the evaluation of the following methods and protocols: a. Eazyplex^®^ MRSA Plus loop‐mediated isothermal amplification (LAMP) assay directly from BC fluid; b. MALDI-TOF MS subtyping on BC pellet extracted with Rapid Sepsityper^®^ protocol and on 4-h short-term subculture; c. Clearview™ Culture Colony PBP2a SA immunochromatography assay on BC pellet and on 4-h short-term subculture; d. EUCAST RAST cefoxitin screen test performed directly from BC and including reading times at 4-h, 6-h and 16–20-h.

**Results:**

Eazyplex^®^ MRSA plus exhibited the best performance, showing 100% sensitivity, specificity, positive predictive value, and negative predictive value, followed by PBP2a SA Culture Colony Clearview assay and EUCAST RAST cefoxitin screen. MALDI-TOF MS subtyping showed the lowest diagnostic accuracy (59.8 and 65.7% directly from BC and from 4-h subculture, respectively). In detail, sensitivity and specificity ranged from 24.3% to 20.4% and from 88.9% to 98.3% for protocols performed from BC pellet and 4-h subculture, respectively.

**Conclusions:**

The Eazyplex^®^ MRSA Plus and the immunochromatographic Clearview™ PBP2a SA Culture Colony methods can provide reliable results within 1 h from the start of positive BC processing. MALDI TOF MS subtyping showed unacceptable specificity by performing analysis from BC pellets, while its sensitivity depends on the prevalence of PSM-positive MRSA strains. The EUCAST RAST, based on disc diffusion, showed excellent performance with a time-to-result of at least 4 h.

## Introduction

*Staphylococcus aureus* is one of the main Gram-positive bacteria involved in a wide variety of clinical manifestations that can affect all ages. Infections are common in both community and hospital settings and their effective treatment is hampered by the emergence of multi-drug resistant strains [1, 2]. *S. aureus* bloodstream infection (BSI) is a serious clinical condition associated with high hospital mortality, long hospital stay and high medical costs [3–6]. In this context, delayed administration of effective antimicrobials has been associated with poor outcomes [7, 8]. First-line antibiotics used for the treatment of methicillin-susceptible S. aureus (MSSA) bacteremia are β-lactams as flucloxacillin and cefazolin. However, methicillin-resistant *S. aureus* (MRSA) has become increasingly prevalent worldwide and causes both community and healthcare-associated infections [9–11]. Methicillin resistance is caused by the expression of an altered penicillin-binding protein (PBP2a) that shows a lower binding affinity to β-lactams. PBP2a is encoded by the *mecA* gene carried on a mobile genetic element, the staphylococcal cassette chromosome *mec* (*SCCmec*) [12, 13]. Furthermore, a new homologous determinant of *mecA*, called *mecC*, has been identified among MRSA, but is rarely found [14].

As MRSA strains are not susceptible to the main β-lactam antibiotics, the most effective treatment options include glycopeptides, linezolid, daptomycin and the new-generation cephalosporins with anti-MRSA activity, ceftaroline and ceftobiprole [15–18]. A recent report showed that empirical antimicrobial therapy was inappropriate in 47% of patients with MRSA bacteremia, and more than 90% of patients suffering from MSSA BSI were initially treated with broad-spectrum empirical therapy including vancomycin or daptomycin [17]. Therefore, the ability to discriminate MSSA from MRSA is essential for appropriate antimicrobial management of *S. aureus* infections [17, 18].

Although it remains the gold standard for the diagnosis of BSI, blood culture (BC) requires at least 48 h starting from positive BC processing to obtain microbial identification and antimicrobial susceptibility test (AST) results. In recent years, several molecular panels have been developed for the diagnosis of BSI directly from positive BC, some of which able to simultaneously detect MSSA/MRSA and many other pathogens within 1–2 h [19]. However, high costs have prevented their widespread implementation in routine BC diagnostic workflows. Other commercially available and lower-cost diagnostic methods for the early identification of *S. aureus* and/or prediction of methicillin resistance include matrix-assisted laser-desorption/ionization time-of-flight (MALDI-TOF) mass spectrometry (MS) analysis [20–22], lateral flow immunoassays [21, 23, 24], and molecular assay targeting both *S. aureus* and *mecA*/*mecC* [20, 25, 26]. In addition, machine learning approaches based on MALDI-TOF MS represent a new frontier for the rapid diagnosis of antimicrobial resistance. This approach consists of the detection of specific peaks in the mass spectrum, which can be associated with the identification of bacterial strains with resistance determinants (e.g. methicillin resistance in *S. aureus* or KPC carbapenemase production in Enterobacterales) [27–29]. Rhoads et al. developed a rapid MRSA detection method based on the specific peak of phenol-soluble modulin-mec (PSM-mec) by MALDI-TOF MS [30]. The PSM-mec peptide is encoded by the *SCCmec* types II, III and VIII in the vicinity of *mecA* [31]. Subsequently, a *S. aureus* subtyping module for MRSA identification based on the PSM-mec peak as an advanced function of the Bruker MALDI Biotyper system without any additional manual operation, time or reagent was developed [32].

More recently, EUCAST developed a rapid AST (RAST) method based on disc diffusion to provide susceptibility results after 4 h, 6 h and 8 h of incubation and including the cefoxitin test for methicillin resistance screening in *S. aureus* [33, 34].

The aim of this study was to evaluate four diagnostic methods to timely identify methicillin resistance in *S. aureus* positive BCs: (i) a loop-mediated isothermal amplification (LAMP) assay (Eazyplex^®^ MRSA Plus, Amplex BioSystems, Giessen, Germany), (ii) the MALDI-TOF MS subtyping (MBT HT Subtyping IVD Module, Bruker Daltonik, Bremen, Germany) (iii) an immunochromatography assay (Clearview™ PBP2a SA Culture Colony Test, Abbott, Illinois, USA) (iiii) the EUCAST RAST cefoxitin screen test.

## Material and methods

### Study design

This study was performed in a tertiary teaching hospital in Italy over a year period (October 2022–October 2023). The BactAlert Virtuo instrument (BioMérieux, Marcy l’Ètoile, France) was used for BC processing during the study period. BCs tested positive to Gram-positive cocci at microscope examination and with reliable identification (score ≥ 2.0) of *S. aureus* by MALDI-TOF MS (Bruker Daltonik) on 4-h-short-subcultures were included. Only one BC bottle per patient/BSI event was included in the analysis and samples collected from patients with *S. aureus* BSI within the previous 30 days were excluded.

Additionally, 36 BCs spiked with *S. aureus* clinical strains, previously characterized for *mecA*/*mecC* carriage status, were included [17, 35]. For this aim, clinical BC bottles (BacT/Alert FA/FN Plus, BioMérieux) that remained negative after five days of incubation were anonymized, spiked and loaded in BactAlert Virtuo instrument (BioMérieux) as previously described [17].

Clinical and spiked BCs were subjected to the evaluation of the following methods and protocols:

a. Eazyplex^®^ MRSA Plus LAMP assay directly from BC fluid; b. MALDI-TOF MS subtyping from BC pellet extracted with Rapid Sepsityper^®^ protocol and on 4-h short-term subculture; c. Clearview™ PBP2a SA immunochromatography assay on BC pellet extracted with Rapid Sepsityper protocol and on 4-h short-term subculture; d. EUCAST RAST cefoxitin screen test performed directly from BC and including reading times at 4-h, 6-h and 16–20-h.

The results provided by the rapid diagnostics methods were compared with those obtained by conventional reference methods.

### Reference methods

Overnight blood-agar subcultures were examined to check the purity, and MALDI-TOF MS on pure colonies was performed to confirm *S. aureus* identification. Subsequently, methicillin susceptibility/resistance was determined on overnight subcultures using the cefoxitin screen test (30 ug) [36], and the Eazyplex^®^ MRSA Plus molecular assay for the detection of *mecA/mecC* genes following the manufacturer's instructions (https://www.eazyplex.com).

### Eazyplex^®^ MRSA Plus LAMP assay

**Fig. 1:**
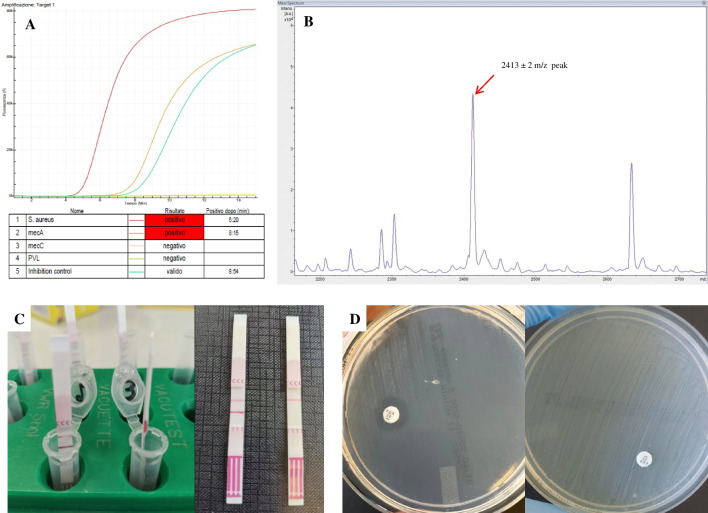
Diagnostic methods evaluated for rapid determination of methicillin resistance in *S.aureus* positive blood cultures. **A**. *mecA*-positive *S. aureus* detected by Eazyplex^®^ MRSA Plus LAMP assay; **B**. presumptive PSM positive MRSA detected by MALDI-TOF MS subtyping analysis; **C**. Clearview™ PBP2a SA Culture Colony immunochromatography assay (positive result is indicated by positivity of both test and control lines); **D**. EUCAST RAST cefoxitin screen test (reading at 6 h): on the left a MSSA (cefoxitin 30 µg inhibition zone ≥ 18 mm), on the right a MRSA (no inhibition zone)

The Eazyplex^®^ MRSA Plus assay is a commercial LAMP assay detecting *S. aureus*, *mecA*, *mecC* and Panton-Valentine leucocidin (PVL-Toxin). The Eazyplex^®^ kits are CE-marked, validated for isolated bacterial colonies and showed excellent agreement with conventional polymerase chain reaction assays [37, 38]. The investigative protocol applied to the BCs included in this study was as follows: 25 mL of BC fluid were mixed with 500 mL of RALF solution provided by the Eazyplex^®^ kit, and boiled for 2 min. After centrifugation at 1000 g for 30 s, 25 mL of the supernatant were added to each tub of the Eazyplex^®^ test strip containing the lyophilized master mix. The strip was gently knocked to remove air bubbles and loaded into the Genie II instrument. Amplification was measured by real-time fluorescence detection using a DNA intercalating dye (Fig. 1).

### Rapid Sepsityper^®^ protocol

The Rapid MBT Sepsityper^®^ IVD kit (Bruker Daltonik) was performed to recover bacterial pellet from BCs following manufacturer’s instructions with some modifications as follows: (i) transfer nominal 1 mL of BC fluid to an Eppendorf tube; (ii) add nominal 200 μL of lysis buffer and mix by vortexing for 10 (± 5) s; (iii) centrifuge the tube for 2 min at 13 000 rpm at room temperature; (iv) remove the supernatant by pipetting and discard; (v) add nominal 1 mL of washing buffer and resuspend the pellet by pipetting up and down; (vi) centrifuge the tube for 1 min at 13, 000 rpm at room temperature; (vii) remove the supernatant from the pellet by pipetting and discard; and (viii) if the bacterial pellet is contaminated with material from the blood, add 1 mL of washing buffer, vortex for 10 (± 5) s to suspend the bacterial pellet, allow corpuscular material to settle for 30 s, transfer the supernatant bacterial suspension into a new Eppendorf tube and proceed to step (vi).

### MALDI-TOF MS subtyping analysis

MALDI-TOF MS analysis was performed on BC pellet, 4-h blood agar short subculture and overnight subcultures following manufacturer’s instructions. For all three protocols, a small amount of bacteria (spin-down pellet, short-term subculture, pure overnight colonies) was transferred onto the MALDI-TOF steel target plate using a toothpick, coated with 1 µL of HCCA matrix, allowed to dry at room temperature and subjected to MALDI-TOF MS analysis on Bruker Microflex LT mass spectrometer. FlexControl 3.3 and Maldi Biotyper 3.0 software (Bruker Daltonics) were applied to acquire the spectra and for the identification of the isolates with the Biotyper module, respectively. The Bacterial Test Standard was used for calibration purposes. Each sample was tested in triplicate and species identification with highest confidence score was recorded. *S. aureus* strains with log (scores) ≥ 2.0 were further automatically identified by the MBT Subtyping IVD Module. When the peak of 2413 ± 2 m/z existed, the software alerted the *S. aureus* strain as “presumptive PSM positive MRSA” (Fig. 1). Positivity was assumed if the specific peak was detected in at least one out of three replicate tests.

### Clearview™ PBP2a SA Culture Colony immunochromatography assay

The test was performed both on BC bacterial pellet after MALDI-TOF MS analysis and on 4-h short-term subculture. The two protocols differed only in the first step: two drops of Reagent 1 were dispensed in the tube containing the BC bacterial pellet, whereas 1 µl loop of fine growth from the short-term subculture was sampled and added to two drops of Reagent 1 in the reagent tube provided by the kit. Then, two drops of Reagent 2 were dispensed and the tube was vortexed until the suspension turned from a blue color to a clear, colorless suspension. The test strip was then added with arrows pointing down. Test results were read by two independent readers after five min of migration at room temperature according to the manufacturer’s recommendations (Fig. 1). Any positivity of the sample band after five min was not taken into account.

### EUCAST RAST cefoxitin screen test

EUCAST RAST was performed according to EUCAST guidelines [39]. Briefly, cefoxitin (30 µg) disc (Oxoid Ltd, Hampshire, UK) was placed on 90 mm Mueller–Hinton agar plate immediately after inoculation and spreading of 150 µL of the BC fluid (Fig. 1). The plate was incubated at 35 ± 1 °C in ambient air. Inhibition zone diameter was read at 4 h, 6 h and 16–20 h and interpretation was performed according to current guidelines [40]. According to EUCAST recommendations, the quality-control procedure was performed to validate the performance of the antibiotic disc, the agar used and the inhibition zone reading methods using *S. aureus* ATCC 29213 [41].

### Data analysis

Descriptive data are presented as absolute (n) and relative (%) frequencies. Sensitivity, specificity, positive predictive value (PPV), negative predictive value (NPV), and accuracy of the rapid diagnostic protocols in detecting methicillin-resistance with 95% CI were computed using MedCalc software version 16.8.4. Comparison involving dichotomous variables was tested using X^2^ test. Statistical significance was set a p-value < 0.05.

## Results

In the study period, 73 out of 76 enrolled clinical BCs revealed to be monomicrobial and were included in the analysis. Considering clinical (*n* = 73) and spiked (*n* = 36) BCs, overall methicillin resistance rate was 44% (34.2% and 63.9% in clinical and spiked BCs, respectively), and *mecA* carriage was documented in all MRSA isolates.

Reliable species identification (log (score) ≥ 2.0) were obtained by MALDI-TOF MS in 75.2%, 93.6% and 100% of the total BC samples using Rapid Sepsityper^®^ protocol, short-term subcultures and overnight subcultures, respectively. The performance of automated detection of PSM-positive MRSA by MALDI-TOF MS subtyping was presented in Table 1. Detection of 2413 ± 2 m/z peak occurred in 24.3%, 25% and 29.2% of MRSA isolates using the Rapid Sepsityper^®^ protocol, short-term subcultures and overnight subcultures, respectively. At the same time, the three MALDI-TOF MS protocols showed false positive results in 11.1% (*n* = 5), 1.7 (*n* = 1) and 1.6% (*n* = 1) of the total MSSA isolates with reliable identification score, respectively. No significant discrepancy was observed according type of BC (clinical vs. spiked) and type of bottle used (BACT/ALERT^®^ FA Plus vs. FN Plus).

**Table 1:** Performance of MALDI-TOF MS subtyping for identification of methicillin-resistant *S. aureus* from blood cultures

		MALDI-TOF MS from blood culture (Rapid Sepsityper Protocol)				MALDI-TOF MS from 4-h subculture				MALDI-TOF MS from pure overnight colonies		
		Score ≥ 2	Score 1.8–1.99	Score < 1.8	Methicillin resistance peak	Score ≥ 2	Score 1.8–1.99	Score < 1.8	Methicillin resistance peak	Score ≥ 2	Score 1.8–1.99	Methicillin resistance peak
Clinical samples (n = 73)	MRSA (n = 25)	18 (72%)	7 (28%)	0	4/18 (22.2%)	23 (92%)	1 (4%)	1 (4%)	3/23 (13%)	25 (100%)	0	5/25 (20%)
	MSSA (n = 48)	35 (72.9%)	10 (20.8%)	3 (6.2%)	5/35 (14.3%)	47 (97.9%)	1 (2.1%)	0	0/47	48 (100%)	0	1/48 (2.1%)
Spiked samples (n = 36)	MRSA (n = 23)	19 (82.6%)	4 (17.4%)	0	5/19 (26.3%)	21 (91.3%)	1 (4.3%)	1 (4.5%)	6/21 (28.6%)	23 (100%)	0	9/23 (39.1%)
	MSSA (n = 13)	10 (76.9%)	2 (15.4%)	1 (7.7%)	0/10	11 (84.6%)	1 (7.7%)	1 (7.7%)	1/11	13 (100%)	0	0/13
Total (n = 109)	MRSA (n = 48)	37 (77.1%)	11 (22.9%)	0	9/37 (24.3%)	44 (91.7%)	2 (4.2%)	2 (4.2%)	9/44 (20.5%)	48 (100%)	0	14/48 (29.2%)
	MSSA (n = 61)	45 (73.8%)	12 (19.7%)	4 (6.6%)	5/45 (11.1%)	58 (95.1%)	2 (3.3%)	1 (1.6%)	1/58 (1.7%)	61 (100%)	0	1/61 (1.6%)
	Total	82 (75.2%)	23 (21.1%)	4 (3.7%)	14/82 (17.1%)	102 (93.6%)	4 (3.7%)	3 (2.7%)	10/102 (9.8%)	109 (100%)	0	15/109 (13.8%)

*MRSA* methicillin-resistant *S. aureus*, *MSSA* methicillin-susceptible *S. aureus*

The Clearview™ PBP2a SA immunochromatography test identified all *mecA*-positive isolates, both in clinical and spiked samples, and both by testing BC pellets and short-term subcultures. Three MSSA positive samples showed false positive results (BC pellet, *n* = 2; short-term subculture, *n* = 1) (Table 2).

**Table 2:** Performance of LAMP assay (Eazyplex^® ^MRSA plus) and lateral flow immunoassay (Clearview^TM ^PBP2a SA Culture Colony test) for the identification of methicillin-resistant*S. aureus*

		Directly from blood culture						4-h subculture	
		Easyplex^®^ MRSA plus				PBP2a SA Clearview (Rapid Sepsityper Protocol)		PBP2a SA Clearview	
		Invalid	*S. aureus*	mecA	mecC	Positive	Negative	Positive	Negative
Clinical samples (*n* = 73)	MRSA (*n* = 25)	0	25 (100%)	25 (100%)	0	25 (100%)	0	25 (100%)	0
	MSSA (*n* = 48)	2 (4.2%)	48 (100%)	0	0	1 (2.1%)	47 (97.9%)	1 (2.1%)	47 (97.9%)
Spiked samples (*n* = 36)	MRSA (*n* = 23)	1 (4.3%)	22 (95.6%)	22 (95.6%)	0	23 (100%)	0	23 (100%)	0
	MSSA (*n* = 13)	0	13 (100%)	0	0	1 (7.7%)	12 (92.3%)	0	13 (100%)
Total (*n* = 109)	MRSA (*n* = 48)	1 (2.1%)	47 (97.9%)	47 (97.9%)	0	48 (100%)	0	48 (100%)	0
	MSSA (*n* = 61)	2 (3.3%)	59 (96.7%)	0	0	2 (3.3%)	59 (96.7%)	1 (1.6%)	60 (98.4%)
	Total	3 (2.7%)	106 (97.2%)	47 (43.1%)	0	50 (45.9%)	59 (54.1%)	49 (44.9%)	60 (55%)

*MRSA* methicillin-resistant *S. aureus*, *MSSA* methicillin-susceptible *S. aureus*

Eazyplex^®^ MRSA plus LAMP assay achieved invalid results in three BCs samples (clinical, *n* = 2; spiked, *n* = 1) and allowed detection of *mecA* in 47 out of 48 (97.9%) MRSA positive BCs. Overall, no false negative or false positive results were observed (Table 2).

The performance of EUCAST RAST cefoxitin screen test was reported in Table 3. The overall rate of readable inhibition zone diameters after 4 h was 97.2% (106/109) and reached 100% after 6 h. Overall, the proportion of results in the area of technical uncertainty (ATU) were 0.9% at both 4 h and 6 h, and 2.8% at 16–20 h. The cefoxitin screen test tested positive in 97.9% of the MRSA-positive samples at both 4 h and 6 h, and in 97.9% at 16–20 h. No false positive results was achieved among MSSA positive samples.

**Table 3:** Performance of EUCAST RAST (cefoxitin screening) for rapid determination of methicillin resistance in *S. aureus*

		4-h				6-h				18–20-h		
		Readable zones	Negative (≥ 16 mm)	ATU (15 mm)	Positive (< 15 mm)	Readable zones	Negative (≥ 18 mm)	ATU (17 mm)	Positive (< 17 mm)	Negative (≥ 22 mm)	ATU (21 mm)	Positive (< 21 mm)
Clinical samples (*n* = 73)	MRSA (*n* = 25)	25 (100%)	0	0	25/25 (100%)	25 (100%)	0	0	25/25 (100%)	0	0	25/25 (100%)
	MSSA (*n* = 48)	46 (95.8%)	45/46 (97.8%)	1/46 (2.2%)	0	48 (100%)	47/48 (97.9%)	1/48 (2.1%)	0	46/48 (95.8%)	2/48 (4.2%)	0
Spiked samples (*n* = 36)	MRSA (*n* = 23)	22 (95.7%)	1/22 (4.5%)	0	21/22 (95.5%)	23 (100%)	1/23 (4.3%)	0	22/23 (95.7%)	0	1/23 (4.3%)	22/23 (95.6%)
	MSSA (*n* = 13)	13 (100%)	13/13 (100%)	0	0	13 (100%)	13/13 (100%)	0	0	13/13 (100%)	0	0
Total (*n* = 109)	MRSA (*n* = 48)	47 (97.9%)	1/47 (2.1%)	0	46/47 (97.9%)	48 (100%)	1/48 (2.1%)	0	47/48 (97.9%)	0	1/48 (2.1%)	47/48 (97.9%)
	MSSA (*n* = 61)	59 (96.7%)	58/59 (98.3%)	1/59 (1.7%)	0	61 (100%)	60/61 (98.4%)	1/61 (1.6%)	0	59/61 (96.7%)	2/61 (3.3%)	0
	Total	106 (97.2%)	59/106 (55.7%)	1/106 (0.9%)	46/106 (43.4%)	109 (100%)	61/109 (56%)	1/109 (0.9%)	47/109 (43.1%)	59/109 (54.1%)	3/109 (2.8%)	47/109 (43.1%)

*ATU* area of technical uncertainty, *MRSA* methicillin-resistant *S. aureus*, *MSSA* methicillin-susceptible *S. aureus*

Comparison of diagnostic performances of the various methods and protocols was shown in Table 4. Eazyplex^®^ MRSA plus test exhibited the best performance, showing 100% sensitivity, specificity, PPV, NPV and accuracy (CI 95% 92.4–100%), followed by PBP2a SA Culture Colony Clearview assay and EUCAST RAST cefoxitin screen.

**Table 4:** Comparison of diagnostic performance of MALDI-TOF MS subtyping, EUCAST RAST, LAMP assay (Eazyplex^®^ MRSA plus) and lateral flow immunoassay (PBP2a SA Clearview) for identification of methicillin-resistant *S. aureus* from blood cultures

	Eazyplex^®^ MRSA plus	MALDI-TOF MS subtyping		PBP2a SA Clearview		EUCAST RAST cefoxitin screen		
		From blood culture (Rapid Sepsityper Protocol)	4-h subculture	From blood culture (Rapid Sepsityper Protocol)	4-h subculture	4-h	6-h	18–20 h
Sensitivity (%) [95% CI]	47/47; 100% [92.4–100%]	9/37; 24.3% [11.8–41.2%]	9/44; 20.4% [9.8–35.3%]	48/48; 100% [92.6–100%]	48/48; 100% [92.6–100%]	46/47; 97.9% [88.7–99.9%]	47/48; 97.9% [88.9–99.9%]	47/47; 100% [92.4–100%]
Specificity (%) [95% CI]	59/59; 100% [93.9–100%]	40/45; 88.9% [75.9–96.3%]	57/58; 98.3% [90.8–100%]	59/61; 96.7% [88.6–99.6%]	60/61; 98.4% [91.2–100%]	58/58; 100% [93.8–100%]	60/60; 100% [94–100%]	59/59; 100% [93.9–100%]
PPV % [95% CI]	100% [92.4–100%]	64.3% [39.8–83.1%]	90% [54.2–98.6%]	96% [86–98.9%]	98% [87.3–99.7%]	100% [92.3–100%]	100% [92.4–100%]	100% [92.4–100%]
NPV % [95% CI]	100% [93.9–100%]	58.8% [53.7–63.8.%]	62% [54.6–73.9%]	100% [93.9–100%]	100% [94–100%]	98.3% [89.3–99.7%]	98.4% [89.6–99.8%]	100% [93.4–100%]
Accuracy %	100% [96.6–100%]	59.8% [48.3–70.4%]	65.7% [55.6–74.8%]	98.2% [93.5–99.8%]	99.1% [95–100%]	99% [94.8–100%]	99.1% [95–100%]	100% [96.6–100%]

Invalid Eazyplex^®^ MRSA plus results, MALDI TOF MS spectra with log (scores) < 2.0 and cefoxitin zone diameters not readable or falling in the area of technical uncertainty were excluded from the analysis

*PPV *Positive Predictive Value, *NPV* Negative Predictive Value

MALDI-TOF MS subtyping showed the lowest diagnostic accuracy (59.8 and 65.7% directly from BC and from 4-h subculture, respectively). In detail, sensitivity and specificity ranged from 24.3% to 20.4% and from 88.9% to 98.3% for protocols performed from BC pellet and 4-h subculture, respectively.

## Discussion

The rapid identification of *S. aureus* and the differentiation of MRSA from MSSA directly from positive blood cultures may provide useful information for the optimization of empirical antimicrobial therapy with consequent impact on clinical outcomes [17, 41, 42].

In this study, we compared the performance of four diagnostic methods for the rapid determination of methicillin resistance in *S. aureus*-positive BCs. The Eazyplex^®^ MRSA Plus LAMP assay was performed directly from positive BC and showed excellent performance with a time-to-result of half an hour. This finding was consistent with previous studies involving Eazyplex^®^ MRSA Plus and/or Eazyplex^®^ MRSA (targeting *S. aureus*, *S. epidermidis*, *mecA*, and *mecC*) tests [37, 38]. In addition, the Eazyplex^®^ MRSA Plus test has the advantage over the other phenotypic methods (e.g. immunochromatography assay or EUCAST RAST) to detect *S. aureus*, PVL-Toxin, and the *mecA* and *mecC* targets. This allows its application on BC samples with suspected *S. aureus* positivity without additional microbial species identification. Furthermore, the detection of PVL-Toxin, a cytotoxin with a high virulence potential of community-acquired-MRSA, provides prognostic insights and indications for application of infection control measures [43]. However, as previously reported, the occurrence of invalid results, probably due to the presence of amplification inhibitors in the blood samples, is the main limitation of commercial LAMP assays when applied directly from positive BC [37, 38]. To reduce the activity of amplification inhibitors, BC samples with invalid results might be diluted and retested with obvious repercussions in terms of increased time-to-result and costs.

The rapid detection of PBP2a using the Clearview™ PBP2a SA Culture Colony Test showed to be a reliable method for the identification of MRSA in our epidemiological context, by testing both from BC pellets and 4-h subcultures. The excellent diagnostic accuracy on short-term subcultures had already been observed in previous studies [44, 45], while our results expanded the knowledge on the application of the assay on bacterial pellet extracted using commercial Rapid Sepsityper^®^ kit, thus significantly reducing time-to-result. The main limitations of the method include the non-detection of PBP2c (encoded by divergent *mecA* homologue, *mecC*) [44, 45], the possibility of false positive results due to polymicrobial cultures including methicillin-resistant coagulase-negative staphylococci [17, 45], and the need to combine the test with a rapid method for microbial species identification (e.g. MALDI-TOF MS analysis or immunochromatographic tests detecting *S. aureus*) [44].

MALDI-TOF MS has now become a widespread method for bacterial pathogen identification in clinical microbiology laboratories [46] and its applications in rapid diagnostics are increasing [28, 47]. The MALDI-TOF MS subtyping analysis offers the ability of predicting bacterial drug-resistant profiles without additional costs and is easily integrated into both conventional and rapid workflows. Herein, we evaluated the automated detection of the PSM-*mec* peak by the MBT Subtyping IVD Module for detection of methicillin-resistance in *S. aureus* clinical isolates. Regardless of the protocol used, the prevalence of the subgroup of methicillin-resistant *S. aureus* harboring the *mecA* cassette containing the gene which encodes the psm-peak related small protein was relatively low (∼ 20–29%). A wide range (from 15 to 61%) of psm-peak positivity has been described in previous studies among MRSA clinical isolates, revealing significant epidemiological differences in the circulation of distinct MRSA epidemic clones [30, 32, 48, 49]. Consistent with evidence aavailable so far, high specificity (> 98%) was observed performing the test on both 4-hand overnight subcultures [30, 32, 48, 49]. Conversely, both lower specificity (88.9%) and PPV (64.3%) were observed performing the test on BC pellets, suggesting occurrence of unspecific peaks in the range 2413 ± 2 m/z using this protocol. A further limitation of the method is the possibility of subtyping analysis only in the case of good quality mass spectra, that correlates with identification confidence score ≥ 2.00. In fact, although the identification rate with a log (scores) ≥ 2.0 reached 100% with the analysis on pure overnight colonies, it dropped to 93.6% and 75.2% with the analysis performed on 4-h subcultures and BC pellets, respectively.

Evidence available so far on EUCAST RAST showed good performance of the method, encouraging its implementation in BC diagnostic workflows in conjunction with MALDI-TOF MS analysis for species identification [34, 50, 51]. In this study, cefoxitin RAST screening identified 98% of overall MRSA, showing only one false negative result at both 4 h and 6 h and no at 16–20 h. In addition, both non-readable and inhibition zone diameters within ATU were very limited.

In conclusion, the Eazyplex^®^ MRSA Plus and the immunochromatographic Clearview™ PBP2a SA Culture Colony methods may provide reliable results within 1 h from the start of positive BC processing by applying the tests directly from BC broth or from the bacterial pellet extracted using the rapid Sepsityper^®^ protocol. MALDI TOF MS subtyping showed unacceptable specificity by performing analysis on BC pellets, while its sensitivity depends on the prevalence of PSM-positive MRSA strains. The EUCAST RAST, based on disc diffusion, showed excellent performance with a time-to-result of at least 4 h. An advantage of the latter is the possibility to in parallel test the susceptibility to other antibiotics commonly used for the treatment of *S. aureus* infections, including erythromycin, aminiglycosides and fluoroquinolones. Further studies are warranted to assess the impact of these methods within fast-track diagnostics workflows on antibiotic management and clinical outcomes.

## Data Availability

Not applicable.
